# The utility of animal models to inform the next generation of human space exploration

**DOI:** 10.1038/s41526-025-00460-5

**Published:** 2025-02-22

**Authors:** Isla Duporge, Talmo Pereira, Santiago Castiello de Obeso, Julius G. Bright Ross, Stephen J. Lee, Allyson G. Hindle

**Affiliations:** 1 Department of Ecology and Evolutionary Biology, Princeton University, Princeton, NJ USA; 2 National Academy of Sciences, Washington, DC USA; 3 Salk Institute for Biological Studies, La Jolla, CA USA; 4 Wu Tsai Institute, Yale University, New Haven, CT USA; 5 Wildlife Conservation Research Unit, Department of Biology, University of Oxford, Oxford, England UK; 6 U.S. Army Research Laboratory, Army Research Office, Durham, NC USA; 7 School of Life Sciences, University of Nevada Las Vegas, Las Vegas, NV USA

**Keywords:** Zoology, Evolution

## Abstract

Animals have played a vital role in every stage of space exploration, from early sub-orbital flights to contemporary missions. New physiological and psychological challenges arise with plans to venture deeper into the solar system. Advances in chimeric and knockout animal models, along with genetic modification techniques have enhanced our ability to study the effects of microgravity in greater detail. However, increased investment in the purposeful design of habitats and payloads, as well as in AI-enhanced behavioral monitoring in orbit can better support the ethical and effective use of animals in deep space research.

## Introduction

Animals have preceded humans in every stage of space exploration, monkeys, apes, dogs, cats, tortoises, mice, rats, rabbits, fish, frogs, spiders, and insects have all been launched as analogs to understand human adaptation to the space environment. Initially focused on survival and safety driven by the race to the moon, after the 1970s, animal research refocused on physiological adaptations in low-Earth orbit (LEO) to better understand risks to human health during long-duration microgravity missions. The international space agenda is now focused on establishing lunar bases as a platform for human crewed missions to Mars, with a transit time of more than seven months each way. Venturing further into our solar system introduces new physiological and psychological challenges for astronauts, in particular, increased exposure to damaging radiation, disrupted circadian rhythms, and more severe sensory deprivation and isolation. The benefits of using animal models to understand adaptations to the space environment are well established, however, a better understanding of how animal models can address challenges exacerbated in long-duration space missions can benefit from further research.

A rich body of research exists on human performance in isolated, confined, and extreme (ICE) environments such as polar expeditions, submarines, and space analogs—simulations on Earth that mimic conditions found in space or on other celestial bodies. This data is a necessary substitute for actual data from space flights; as of 2024, fewer than 700 people have been in LEO or beyond, and very few have been in orbit continuously for over a week. In human studies, sample sizes are small, whether in simulation or in orbit, and the degree of experimentation is restricted due to logistics as well as safety; consent is required at each step, and the cost of implementing these studies can be very high. Using animal models mitigates some of the challenges associated with astronaut-based studies by providing larger sample sizes over the same time period. Animal-based studies allow for more extensive experimentation, such as exposure to space exploration hazards like radiation, at a lower cost compared to long-duration human analog studies on Earth. While the sample sizes in space-based animal research are still small compared to those in ground-based studies, these limitations in sample sizes and costs could be addressed by increasing industry focus on the purposeful design of habitats and payloads. Animal-based studies are not a substitute for human trials, particularly for studies of psychosocial processes; however, we are on the same neurobiological continuum as other species, and similar biological mechanisms have evolved due to exposure to similar selection pressures. The ethical use of animals to better understand the impacts of space flight can ameliorate some of the most pressing challenges to make long-duration missions returning to the lunar surface and onto Mars possible.

## History of animals in orbit

Animals sent into orbit during early space exploration helped define the physiological risks to humans in this novel environment^1^. Initially, animal models were primarily used to test vehicle safety and survivability, with minimal biological investigation. However, space-based animal research has become increasingly sophisticated with advancements in engineering and the enhanced understanding of genetics, enabling a better understanding of human biology through animal studies. With the first deployment of dogmonauts Dezik and Tsygan in 1951, species choice became a salient element of space biology. While the Soviet space program employed dogs as their preferred model (specifically using strays under the presumption that they were hardier and thus better equipped to handle the stress of the space program), the United States preferred primates due to their greater taxonomic proximity to humans^2^. The squirrel monkey Gordo was the first primate in space in 1958, reaching an altitude of 500 km before suffering a parachute malfunction on re-entry and disappearing into the South Atlantic Ocean^3^. Despite the loss, the mission was considered a success, and numerous primates, including rhesus monkeys, chimpanzees, and numerous macaque species, were subsequently sent beyond the Kármán line^2^. Since the 1950s, significant advancements have been made in the translatability of animal-based research. Comparative genomics has revealed that many species share functional DNA elements with humans^4^, thereby broadening the selection of model species. Once animal models successfully demonstrated the survivability of space missions for short periods, attention shifted to longer-term missions, and animals became a mainstay of space biology throughout the space race of the 1960s.

The calming of the Cold War led to an agreement on ‘Cooperation in the Exploration and Use of Outer Space for Peaceful Purposes’ between the United States’ National Aeronautics and Space Administration (NASA) and the Soviet Space Program in 1972. Over the following 23 years, eleven Cosmos/Bion satellites were launched, with flight durations between 5–22 days. Payloads included tissue cultures, plants, animals, bird eggs, molluscs, newts, fish, fruit flies, rats, and primates^5^. These eleven Bion missions remain the most extended collaborative biological space research project in space history and the most extensive data source on how living organisms function in microgravity. From these flights, important discoveries were made on how space radiation impacts human organs, and it was shown that fertilization and embryonic development of mammal and bird eggs is possible in microgravity^6^. Plans for the twelfth Bion mission ended abruptly in 1997 when one of the rhesus monkeys on Bion 11 died under anesthesia one day after return^7^. Prior to Bion 11, anesthesia had not been administered within seven days of return, giving the subjects’ bodies time to adjust to the Earth’s environment. As a result of the event, NASA changed the procedures it would use to treat astronauts post-flight, but the monkey’s death also signaled the end of the Bion missions and the use of primates as a life science subject in space^1^.

The next significant life science mission was STS-90 Neurolab Spacelab, a 16-day shuttle flight launched in 1998, which included around two thousand animal subjects, including rats, mice, snails, crickets, and several fish species used to understand better how microgravity impacts the brain and central nervous system^8,9^. One of the payloads contained 76 rats used for numerous experiments, including survival surgery - primarily anesthesia, wound closure, and wound healing. This allowed astronauts to test if they could control surgical fluids and instruments in microgravity – no decrements in manual dexterity were found despite operative time being slightly longer, demonstrating that it would be feasible to carry out dissections and surgical procedures on humans in microgravity if necessary^10^. During the STS-90 mission, a veterinarian astronaut conducted these intricate procedures, and the crew received extensive pre-launch training in animal care and in-flight handling techniques. This comprehensive training ensured they were well-prepared for the mission’s biological experiments. Although the variety of species that can be used in scientific experiments has been restricted due to well-warranted increases in ethics standards since the 1980s, payloads carrying model organisms are still relied on to understand the biological risks of the space environment - helping to pave the way for longer-duration missions^11^.

## Animals in orbit since 2000

When the study of larger mammals ceased, research shifted toward using smaller organisms. Despite being taxonomically further from humans, smaller model organisms provide many advantages. The roundworm (*Caenorhabditis elegans*), the fruit fly (*Drosophila melanogaster*), and the mouse (*Mus musculus*) are all common models, all of which have their complete genome sequenced. The optimal model organism to use in an experiment depends on the scientific questions being asked, the amount of crew time available to manage experiments, and space available, which is a sharply constrained resource in space missions. Smaller organisms such as *C. elegans* and *D. melanogaster* do not require manual feeding, depend on limited upkeep, and occupy small spaces^12^. The ability to conduct experiments on large numbers of these model organisms also provides the necessary sample sizes and experimental power to understand space-induced changes, particularly regarding physiology. Advances in gene editing techniques, knock-out capabilities, and knowledge of the genome have increased the translatability of research on small model organisms to understand the biological impacts of spaceflight stressors on human biology^13^.

C. *elegans* has a large brood size and a three-week lifespan, is easy to culture in challenging environments, and shares various evolutionarily conserved mechanisms with a wide range of animals. Consequently, it has been used as a model organism to understand the molecular mechanisms regulating adaptations to microgravity since the space shuttle era^14^. In 2005, the Columbia STS-107 mission ended in tragedy when the shuttle disintegrated on atmospheric re-entry, resulting in the loss of the crew - a few days later, several containers of *C. elegans* were found alive^15^, demonstrating the resilience of these organisms and payload design to impact.

*D. melanogaster* was one of the first organisms to be launched into space and has since been used to generate significant findings in neurobehavioral, aging, immune, cardiovascular, developmental, and multi-omics changes across tissues and developmental stages^16^. The average lifespan of an adult fly is approximately 60–80 days, with females laying hundreds of eggs a day, resulting in significant population sizes for study, enabling statistically significant measurements of physiological responses to the spaceflight environment to be made. Different lines of these model organisms have been flown, ranging from wild-type strains to specific mutants targeted to the biological question addressed by the science payload. Due to its small size and ease of breeding, *D. melanogaster* can be loaded onto spaceships in large numbers even when astronaut participation is limited or non-existent^17^.

C-CRISPR, a refinement of the CRISPR-Cas9 gene-editing technology, has brought significant advancements in the use of animal models for scientific research, enabling precise genetic modification and reducing off-target effects that can complicate experimental results. This increased accuracy is essential for creating animal models that faithfully replicate human diseases. C-CRISPR facilitates the creation of chimeric and knockout models with multiple genetic modifications^18^, which enables a closer study of the effects of microgravity on bone density and muscle atrophy and how the spaceflight environment impacts developmental processes and immune function. Advances in knockout models enable insight into diseases like cancer, which is of critical concern in the space environment^19,20^. While chimeric models, where cells from different organisms are combined, can help reveal the mechanisms behind how radiation in the space environment damages DNA^21^. These advanced animal manipulation capabilities offer essential tools to better understand biological processes and aid the development of therapeutic strategies to support the next generation of human space exploration. An important consideration in all experiments is how long the observed physiological adaptations to microgravity persist after an organism returns to Earth in order to assess whether the effect is short- or long-term. Another consideration is the elevated mortality risks for animals in orbit compared to ground experiments. Given the higher cost of space experimentation, it is essential to minimize this risk as much as possible. One study investigating circadian rhythms using *D. melanogaster* found 40% of space-flown flies survived, compared with 60% and 62% in mirrored ground-based experiments. For further details on the use of *C. elegans* in spaceflight, see ref. ^12^, and for *D. melanogaster*^16^.

Sending these model organisms into orbit over the last few decades has enabled a finer understanding of several physiological challenges in the space environment. Challenges include space radiation^22^, oxidative stress^23^, increased cortisol levels^24^, disruption of circadian rhythms^17^, immune modulatory changes^25^, dysregulated metabolism, mitochondrial dysregulation, epigenetic/regulatory changes, shifts in host-microbe interactions, and weakened muscular and bone health^26^. Wound and fracture healing is slower in microgravity, and immunity is weakened; furthermore, the confined environment on spacecraft leads to a greater risk of microbial contamination and infection due to recycled air and water and limited personal hygiene.

The NASA Human Research Program has highlighted spaceflight hazards that pose physiological challenges to astronauts^27^. Traveling away from the protection of the Earth’s magnetosphere exposes astronauts, as well as animal study subjects, to a constant background of galactic cosmic radiation as well as solar particle events. Long-term carcinogenic impacts of heightened radiation exposure during flight are a crew health concern, along with potential acute alterations to neurological, cardiovascular or immune function, making radiation exposure one of the primary health concerns for human presence in space^22,28^. Dose-effect models are essential to optimize protective measures for long-duration space missions^29^. Animal models have been critical to developing the datasets that could establish radiation schedules for long-duration crews and possible sources of protection^30^. Spacecraft environments offer low light levels and artificial light: dark cycles that may not conform to the 24-h circadian cycles experienced on Earth. This space environment can disrupt sleep homeostasis and circadian rhythms^31,32^ via altered lighting. Such disruptions negatively affect crew mood, performance, and health, for which environmental or physiological interventions are sought^33^. Animal models have enabled a better understanding of circadian oscillators at behavioral, physiological, and genomic levels in standard gravity, and these models provide insight into changes in the space environment.

An important point regarding such research is that all animal research in federally funded institutions in the United States is tightly regulated and can only be conducted under appropriate veterinary and committee oversight. Similar restrictions and oversight for animal-based research also apply to other nations’ space agencies. NASA has developed various specific organizational oversight and agency-wide policies and requirements throughout the years, and all research All biomedical animal research in U.S. federally funded institutions, including NASA, is strictly regulated and requires veterinary and committee oversight. Similar policies govern other nations' space agencies. NASA ensures compliance through agency-wide regulations, including NPR 8910.1D^34^, under the oversight of the Chief Health and Medical Officer and adherence to Institutional Animal Care and Use Committee (IACUC) regulations.

## Psychological challenges

Various space agencies and private enterprises are planning long-duration missions to Mars. The expected crew size for these missions is small, and a round-trip would take more than a year^35^; thus, the compromised health of just one crew member could endanger mission success. How astronauts will psychologically cope with being isolated and confined for increased durations and distance from Earth remains unknown. The psychological challenges of long-duration spaceflight are as significant for mission success as physiological challenges, and neither are mutually exclusive. Mood symptoms, including stress, anxiety, depressive episodes and obsessive-compulsive behaviors, have been widely manipulated and modeled in animals under terrestrial conditions^36,37^. Many of these studies can be viewed through the lens of homologous triggers for the human and animal models, in those cases where they share a biological mechanism^38^. For instance, aggression is based on triggers with an evolutionary underpinning such as resource competition, which is heightened in close confinement with others and leads to aggressive behavior^39^. Self-mutilation is found in both humans and animals when subject to stressful situations such as crowding, isolation, separation, and confinement—particularly when these situations are perceived as uncontrollable^40,41^. Separation anxiety is a precursor to depression in both humans and non-humans^42^. As in humans, when infant primates are separated from their mothers, they exhibit an initial stage of protest followed by agitation and sleeplessness^42^. Triggers that give rise to certain emotional states can be studied in animals and, in some cases, can be easier to study than in humans due to the possibility of larger sample sizes and more experimentation being possible. Symptoms of insomnia, irritability, anger and anxiety have all been recorded in space flight and can be better understood through harnessing research on animal models^43^. While analogue studies are valuable, the sample sizes are necessarily small, as setting up experiments where people are voluntarily confined for extended periods is logistically, legally, ethically, and financially challenging.

### Impacts of prolonged confinement and social isolation

Significant inter-individual differences exist in responses to confinement and isolation^44^. In both animal and human societies, social isolation is used as punishment. At the same time, in human society, forms of devotional isolation are practiced worldwide, e.g., monastic living^45^, Khalwa in Islam^46^, Anchorites^47^, etc. The reason for isolation shapes perspective and, thus, experience; studies on anxiety show individuals exposed to the same stimulus, objectively undergoing the same experience, often draw different interpretations and, therefore, differing behavioral responses^48^.

Human responses to different situations are not easy to predict because there is wide variation in interpretation. Preferred amounts of time alone vary across ages, genders and cultures, and there is a complex relationship between physical isolation and perceived loneliness, as the latter is an emotional state that can also be experienced in the company of others^49^. There is a wealth of research on the impact wrought by a dramatic increase in social isolation during the COVID-19 pandemic, which saw large swathes of the human population isolated for extended periods^50,51^. The value of the COVID-19 pandemic in terms of understanding the impacts of social isolation and confinement to inform spaceflight research has been analyzed^52^, and it is known that isolation causes considerable effects on brain structures involved in cognitive functions, including learning and memory formation, spatial navigation, self-control, planning, problem-solving, and emotional control. Sensory monotony is a significant risk in long-duration spaceflight - Earth-based space analogs act as simulations of long-duration space missions used to study the impacts of such monotony^53^. There have been many analog experiments over the last two decades, with Mars500 in 2011 being the most extended experiment to date, during which six international crewmembers were isolated for 520 days^54^. Research missions in Antarctica have also been used as a simulation environment to assess how astronauts could respond to isolation and sensory monotony for extended periods^55,56^.

Although the subjective experience of isolation is unpredictable, several consistent physiological responses to isolation have been found during space analogs: emotional dysregulation, cognitive dysfunction, disruption of sleep-wake rhythms, visual phenomena, and significant changes to body weight^57,58^. One commonly used animal model for exploring social isolation is rearing rodents post-weaning with no handling and monitoring behavior^59^. These studies have revealed that hippocampal function is compromised following isolation—resulting in impaired attention^60^, impaired long-term social memory^61^, and anxiety and anhedonia-like symptoms^62^. Isolation is a stressor that results in alterations in reactivity, social behavior, neurochemical and neuroendocrine system function, and anatomical and behavioral changes^63^. A multimodal assessment of isolation from post-weaning to adulthood and resocialization is needed to better understand the triggers for these physiological effects^64^.

### Small-group dynamics

While isolation is important to understand in support of deep-space exploration, astronauts on a mission to Mars are expected to be in small groups of 3–6^65^. To understand how to encourage harmonious small-group dynamics during these long-term missions, research has focused on assessing the individual attributes that support teamwork. Experiences of depression, anxiety, and anger are heightened when there is low social coherence in a group^56^; thus, during astronaut selection, certain personality traits are screened for^25,66,67^. Research using the ‘Big Five’ model of personality suggests low neuroticism (high emotional stability) and high agreeableness are vital traits for astronauts, while data on conscientiousness, openness to experience, and extraversion are contradictory; thus, it is believed having members with varying levels of these traits may be optimal while extremes at the ends of the scales are avoided^68^. During the 520-day Mars analog mission, negative interpersonal interactions correlated with high anxiety levels^69^, and results from a systematic review of research teams living and working together showed during more extended missions, team members generally spent less social time together than on shorter missions, possibly as a conflict avoidance method^70^. The contrary was found in a model organism experiment where, during a 30-day space flight with 45 mice onboard, increased aggregative behavior (huddling contact) was found compared with identically-housed ground controls^71^. This behavior may be indicative of increased sociality or adaptive thermoregulatory behavior as a reaction to cold stress which is exacerbated in rodents on orbit^72^.

This method of closely monitoring social behavior in orbit compared with ground control can help highlight the impacts of microgravity on interaction dynamics. After the 45 mice returned from the 30-day flight, profound deficits in vestibular and motor responses were seen: reduced voluntary wheel running, avoidance of open areas in open-field tests, and signs of hyperactivity and persistent anxiety-like behaviors compared to identically housed ground controls on earth. However, performance improved in many tests carried out 7 days after return to earth’s atmosphere, showing readjustment is possible^71^.

High genetic diversity in model organisms should be encouraged to reflect human diversity^73^. Depending on the specific scientific research question, genetic diversity within a single experimental group (of a single species) may be preferable to using multiple experimental groups, as the latter would require repeated iterations of the same experiment, thereby increasing cost and amount of time in orbit. While including genetic diversity in one group would be less demanding on resources, this is challenging if pheromone separation is necessary to avoid intergroup behavioral influence, given the small size of habitats^74^. However, more purposeful design of animal habitats to support increased genetic diversity in model organisms can mitigate these constraints, and the industry is encouraged to design such facilities. While analog studies are extremely valuable, the sample sizes are small, and thus, experimental power is weak. Unpredictable patterns of group behavior in humans, even when just one individual changes, make it challenging to collect data that can be used in a predictive sense^66^. Experiments on model organisms can greatly complement analog studies by monitoring objective physiological responses to confinement with large sample sizes and countermeasure preclinical testing to prevent adverse effects of treatments in animal models before strategies are tested in humans.

### Using animal behavior biomarkers to understand psychiatric symptoms

There is a significant similarity between forms of underlying brain mechanisms and emotional expression across species; for example, fear nearly universally triggers the widening of the eyes, heavy breathing, shifts in attention, redistribution of blood, the release of hormones, and freezing^75^. There are well-established behavioral readouts for fear, aggression, and anxiety, but many emotional states require increased research to elucidate underlying mechanisms. However, more salient features of different states, particularly psychiatric diseases, are highly heterogeneous and lack objective tests and validated biomarkers^37,76^. Much research has gone into trying to characterize post-traumatic stress (PTSD) symptoms in animal models, for instance by restraining animals for several hours, followed by forced swim sessions and ether anesthesia. The surge in cortisol simulates exposure to a traumatic event; however, the underlying neurobiology is still not characterized, and triggers for the onset of symptoms in PTSD vary widely in humans^77^.

Humans neurobiologically diverged from other species during evolution, developing more complex genetic architecture, and many psychological states cannot be fully recapitulated in animal models; as a result, the biomedical industry has recently begun to retreat from investing in using animal models to understand more complex human brain disorders due to high failure rates in clinical trials^36^. For example, schizophrenia is a complex psychiatric condition characterized by states of psychosis containing delusions (persistent and sometimes odd ideas) and/or hallucinations (percepts without stimuli). Genome-wide association studies have revealed a significant overlap between the molecular architecture of schizophrenia and human accelerated brain regions unique to our species^78^. Even if the symptoms of schizophrenia are not directly measured in non-human animal models, these have led psychiatry to understand the underlying mechanisms of psychosis^79^. For instance, psychosis is characterized as an aberrant state of salience^80^ where unimportant stimuli become important. This aberrant perception is projected upward through the perceptual-cognitive hierarchy to produce higher-level aberrant cognition^81^. Multiple classic learning phenomena, such as latent inhibition, conditioned inhibition, kamin blocking, and Extension, are well understood in animals and have translational power in humans.

The likelihood of severe psychiatric conditions arising in astronauts during long-duration space flights is unknown and is difficult to quantify. Collecting this data is complicated as less than 700 people have been into space, and this data is held by numerous national space agencies. There are several systematic reviews on research on aircraft pilot mental health^82–84^, some challenges parallel astronauts regarding stress and the need to adapt circadian rhythms.

In pilots, there is a reluctance to disclose mental health deterioration due to the risk this can pose to job security^85^, which is also applicable to astronauts. Avoiding severe psychiatric events on orbit is critically important, considering the rigid chain of command and low-redundancy implicit in many space mission crews. A delusion in one crewmember could result in rapid consequences for the rest of the crew. The potential for an individual to develop neuropsychiatric disorders results from a complex interplay of genetic, environmental, and developmental factors. During astronaut pre-selection, individuals are de-selected if there is any personal or family history of psychiatric risk^86^. However, the probability of these states emerging spontaneously during a long-duration return mission to Mars is not insignificant. Daily stress is an important predictor for developing psychotic symptoms^87^ and isolation is implicated in psychosis^51,88^. While several psychological states, such as depression and OCD have well-characterized signs of behavior, schizophrenia demonstrates how much more complex characterization can be. Schizophrenia is a condition of false inferences or beliefs under the notion that the brain is a statical organ that builds the best hypothesis to predict sensations^89^. Thus, distinct components within the perceptual-cognition hierarchy are disrupted. Hallucinations are produced with excess top-down predictions on sensations, and delusions emerge from aberrant bottom-up prediction errors^81^. The ability to measure this in non-human animals has been approximated through classical conditioning phenomena. For example, animals induced into psychosis-like states using dopamine agonists exhibit learning from irrelevant stimuli, displaying behavioral patterns similar to those observed in patients. The same conditioned-hallucination measurements –percepts occuring in the absence of stimuli or false alarms—have also been observed and mice^90^. Thus while schizophrenia is conceptually and phenomenologically, complex to study, neurobiological and behavioral research on its symptoms (e.g., hallucinations and delusions) can be studied using animal models^91^.

While more extreme states of perception are very complicated to objectively characterize in non-humans several fundamental emotional states in animals can be seen through posture and expression and are similar to those seen in humans such as happiness, sadness, disgust, fear, anger, and surprise^92,93^. In confinement after sustained sensory isolation, humans and animals exhibit stereotypy - a fixed motor ritual resembling obsessive-compulsive behavior^94^. Approximately ten days after launch, young mice in the International Space Station’s (ISS) Rodent Habitat, were observed exhibiting a circling behavior that might be consistent with stereotypy, although none of the validation mice displayed overt physiological signs of chronic stress^11^. However, circling may not be stress-induced, instead, this behavior could serve as a method to reduce anxiety or as a thermoregulatory behavioral response to cold stress^72^. Additional research is required to accurately interpret this behavioral response and determine whether it persists once cold stress is alleviated. Sustained sensory isolation after confinement triggers several symptoms found in both human and animal models, such as increased tonic sympathetic tonus, hypothalamic-pituitary-adrenocortical activation, decreased inflammatory control and immunity^95^, reduced sleep quality^96^, and increased expression of genes regulating glucocorticoid responses^97^. While research looking at spaceflight pathology has predominantly focused on detecting the development of clinical signs due to spaceflight hazards, there has been a recent shift toward countermeasures at the preclinical stage^98^. Animal models are used to understand the nature of spaceflight hazards and the pathologies that hazards induce with a goal to develop effective countermeasures in advance of onset.

As preparations are made to send humans further out in our solar system and for longer periods, we can responsibly utilize animal models to understand neurobiological and behavioral adjustments. To prepare for long-duration space flight, objective physiological markers of behaviors resulting from confinement can be better understood by advancing animal models of isolation and increasing studies on orbit to test how microgravity conditions alter these responses^97^. Significant methodological development has been made in measuring and quantifying behavior using artificial intelligence, which now provides historically unmatched opportunities for advancing our understanding of emotional expression via behavioral observations on and off orbit.

## Using AI to detect fine-scale behavioral features in animal models

The ability to discern subtle behavioral changes between individuals on and off orbit is enhanced by using AI to analyze fine-scale behavioral features in animal models, enabling the discovery of new behaviors and the detection of subtle differences undetectable to the human eye^99^. Behavior comprises a remarkably well-integrated representation of animals’ biology that reflects overall neural and physiological states, including complex internal states such as emotions^100^. This richness has classically been a double-edged sword: the more complex the behavior, the harder it is to tractably extract meaningful quantities that describe it; for example, even if it contains much more information, the nuanced kinematic trajectories of the animal’s entire posture is much harder to quantify and interpret than lever pressing. While this type of reductionism has been prevalent in behavioral testing paradigms for decades, fields like neuroscience have begun to move toward more naturalistic paradigms in which behaviors that animals more likely evolved to produce are prioritized over those that are highly unnatural but easy to measure^101^. This strategy is of particular importance in space biology where, while the environments are decidedly unnatural, it is even less likely that we would know precisely what behaviors to measure and thereby would greatly benefit from using unbiased approaches sensitive to subtle and complex changes in behavioral patterning.

The move towards more naturalistic and comprehensive behavioral quantification, sometimes termed ‘computational ethology’, has been largely enabled by the technological advances of the last decade in computer vision and machine learning^102–104^. In particular, the development of deep learning for markerless motion capture with tools like SLEAP^105,106^ and DeepLabCut^107^ has transformed the landscape of behavioral quantification by making it possible to extract articulated poses from conventional videography without the need for specialized imaging setups or intrusive physical markers. These systems have the potential to capture valuable behavioral readouts for space research: the richness of the postural dynamics enables robust and automated behavioral segmentation (i.e., “scoring”)^108,109^; tools adapted for pose tracking in 3D provide an essential capability for behavioral study in low gravity environments^110–112^; specializations of these systems to facial expression capture^113^ are of high relevance to studies on how affective states evolve in spaceflight and their impacts on performance; and compatibility with real-time operations enable continuous monitoring and advanced experimental paradigms^106,114,115^. Analysis of acoustic communication further complements video-based behavioral monitoring, providing further insight into animal behavior. This is now possible using AI-powered methods to identify individual vocalizations, classify call types, and uncover hidden states and temporal structures within vocal sequences^116–118^.

A crucial opportunity would be to use this technology to analyze the large archive of existing video data available of all rodent research missions that have visited the International Space Station to date, an effort enabled by the NASA GeneLab and Open Science Data Repository (OSDR) which afford access to historical datasets^119^. We caution that the reusability of historical data will be limited by the capabilities of the data capture systems. Deployment of modern behavioral monitoring systems introduces technical challenges^120^. For example, continuous monitoring is demanding on the capabilities of typical data storage and management infrastructure due to the high bitrate of video streams. Moreover, the need for multi-camera systems for 3D analysis exacerbates these issues and introduces hard requirements for synchronization and camera calibration; multi-animal systems where identities of visually indistinguishable animals are essential may require visual markers or independent systems like radio-frequency identification (RFID) tags. These present challenges in resource-constrained environments such as spaceflight and will require significant advances in optimal power and data usage in animal monitoring systems. Nevertheless, recent advances in computing aboard the ISS with the Spaceborne Computer-2^121^ promise breakthroughs that will enable greater capacity for deep learning methods in situ.

Despite these challenges, existing studies on rodent behavior in space have established a foundation for understanding how animals adapt to microgravity. By observing mice in a rodent habitat on the ISS, previous studies reported increased activity levels and the emergence of a unique 3D “circling” behavior^11,122^. This required manual scoring by trained observers, making it intractable to score every class of behavior and larger datasets due to the intensive labor requirements. Others have investigated mouse adaptation to hypergravity and microgravity, observing initial reduced activity followed by recovery, likely due to vestibular system adjustments^123^. By adapting coarser measures of activity (e.g., motion energy) manual labor is minimized, but this sacrifices individual subject identifiability and richer readouts beyond binary (active/inactive) labels. These studies highlight the complexities of monitoring animal behavior during spaceflight and emphasize the importance of considering habitat design for compatibility with modern behavioral quantification technology rather than co-opting those originally designed for welfare and health checks^11^.

Future work looking at animal behavior in spaceflight needs to address these issues, and industry should be encouraged to build purposely designed habitats that better support animal research in space with validation compatibility with AI systems. For example, to adequately detect changes in animal behavior, habitats should be designed with extensive camera coverage to ensure all movement is captured and animal identities can be reliably recovered; that cameras are synchronized and timestamped; that excretions are not accumulated over mission time, limiting visibility^11^; and that the camera positions are well calibrated to enable 3D triangulation of kinematics^112^. Enabling the capabilities afforded by AI will require considerable enhancements to the technological readiness level of behavioral monitoring systems, including the development of efficient machine vision cameras, acoustic recording, data storage, and computational processing hardware. Investments in these capabilities will support both animal research and crewed flight missions. Multimodal integration of behavioral and physiological data (e.g., heart rate, muscle activity, hormone levels) would allow for exploring the physiological mechanisms underlying behavioral adaptations to spaceflight; once validated, these can also serve as automated systems for early detection of psychiatric and neurological impairments (e.g., stress responses or emotional duress) that would otherwise be catastrophic for crew performance if left unmitigated. Realtime and onboard processing of behavioral data can enable more advanced experimental paradigms and relieve the burden on crew time by reducing the need for manual animal welfare checks. Finally, the integration and automation of these technologies will be essential for developing “self-driving labs” and autonomous biomonitoring^124,125^.

## Summary and outlook

### Past and future use of animal models to understand adaptations to the space environment

Animals have long been used in spaceflight exploration; while the initial focus was on whether humans could survive microgravity conditions, the shift has since moved to understanding how to remediate the damaging physiological impacts of being in the space environment for extended periods. The international space agenda is currently focused on establishing lunar bases as a basis for human-crewed missions to Mars, with a transit time of more than seven months each way; this introduces significantly elevated physiological and psychological challenges, in particular, extended sensory deprivation and isolation. The benefits of using animal models to understand adaptations to the space environment have been historically established, and animals continue to be essential to understanding biological challenges that will become more pronounced on longer-duration space missions. It is important to note that many physiological changes observed during spaceflight are either reversed or minimized upon reentry to Earth; separating on-board challenges from longer-term challenges is necessary to understand the level of risk involved. Advances in genetics and the sequencing of *D. melanogaster* and *C. elegans* represent a significant advance in using animals to understand adaptations to the space environment on orbit. *C. elegans* and *Drosophila melanogaster* represent cheap, accessible, transportable model organisms that can be used in large numbers to provide the necessary experimental power to understand changes in physiology^126,127^.

Although an important focus of animal use is the fidelity with which non-human models recapitulate human physiology, key gains in understanding may also arise from studying animal models with natural protection. For example, the physiology of metabolic control and even hibernation has emerged as a medically relevant phenotype that could protect long-duration crews by reducing risks associated with limited nutritional, life support, and psychosocial resources available in flight^128,129^. Organisms capable of profound metabolic depression exist across the tree of life, but importantly, this phenotype is also distributed across the mammalian phylogeny, including in certain primates. Small-bodied rodent hibernators tolerate a range of body temperatures during hibernation and dramatically reduce vital rates. Despite extended periods of inactivity during the hibernation season, animals emerge with relatively little muscle atrophy^130,131^. Reduced metabolism and elevated DNA repair mechanisms are also thought to play a role in naturally occurring radiation resistance^132^. Future investigations could investigate this physiology in space environments in natural hibernators or in induced (“synthetic”) hibernation in model rodents^133^. Although animal models are typically selected for their ability to replicate human physiology; extremophiles also offer unique insights that extend beyond conventional models. For example, the tardigrade (*Tardigrada*) can withstand the vacuum of space and extreme levels of ionizing and unfiltered solar radiation, including ultraviolet-A and ultraviolet-B^134^. The extraordinary resilience to the hazards of the space environment has made it a valuable subject for both past and future research^135^.

### Logistical considerations for future animal experimentation on orbit

Logistical considerations and experimental design in microgravity present unique challenges. As discussed above, monitoring animal welfare in a microgravity environment is challenging and must rely heavily on behavior, as many visual markers (coat condition, temperature) are affected in space. The Committee on Space Research (COSPAR) enacted guidelines on all vertebrate animal research in flight and supporting ground-based (control) studies, requiring, among other things, that all activities receive ethical approval (for example, via the NASA Flight Institutional Animal Care and Use Committee). Housing for animal subjects in space requires specialized hardware that is ventilated but enclosed to contain allergens and other biohazards. Housing enclosures are opened and cleaned only on return to Earth; therefore, food and water provision, handling, and experimental manipulations must be accomplished through glovebox access, providing potential experimental design limitations not present in standard vivarium conditions. These challenges can be overcome with more focused effort on designing and testing animal care/test facilities for astronaut use on orbit.

Recent advancements in spaceflight hardware onboard the science lab on the International Space Station have enabled capabilities for more advanced video graphics analyses, environmental controls, and real-time data telemetry to study model organisms in more detail. At the same time, the growth of commercial aerospace capabilities means there is an increased opportunity for space-based science. Researchers have the ability to repeat experiments, accommodate sufficient sample sizes, and test different hardware designs that are suitable for specific biological applications. With careful experimental design and improved AI-supported monitoring systems, closer observation of various animal behavioral phenotypes is possible. Bespoke solutions are needed for each animal model to achieve high-resolution data collection, particularly for behavioral studies. Moreover, many hardware solutions are modified from existing space-flown hardware due to high costs, which create limitations. However, an increasing number of private space stations are being created, which provides scientists with an opportunity to re-work what science racks look like, and different experimental designs can be iteratively modified so experiments are optimized.

### Recommend areas of future research

Astronauts represent significant financial investment, as do the spacecraft and payloads they operate. Prolonged weightlessness means impacts such as muscle atrophy, bone loss, cardiovascular deconditioning and radiation exposure will become more severe during longer-duration missions. The use of animal models to better understand these physiological impacts is required to make a crewed mission to Mars possible. Greater investment and ingenuity are needed to minimize the logistical constraints in running animal experiments on orbit. However, we are at a point in history when this is becoming feasible as the frequency, safety, and costs of launches has improved. Advances in behavior observation techniques, now allow us to refine animal models for specific research questions, using reliable AI-supported behavioral monitoring in orbit alongside mirrored experiments on Earth. Both the physiological and psychological impacts of extended confinement must be better understood to prepare for long-duration space flight. Behavioral testing has proven useful in identifying underlying triggers for the onset of psychotic conditions. We propose expanding the use of animal behavior models to enhance our understanding of these risks in long-duration spaceflight. Further extended experiments comparing group responses to individual responses in isolation using animal models are warranted to identify parrallels with human behavior. Additionally, expanding research on hibernators and extremophiles may provide valuable insights applicable to human space adaptation.
